# Out-of-pocket expenditure and its determinants in the context of private healthcare sector expansion in sub-Saharan Africa urban cities: evidence from household survey in Ouagadougou, Burkina Faso

**DOI:** 10.1186/s13104-016-1846-4

**Published:** 2016-01-21

**Authors:** Idrissa Beogo, Nicole Huang, Marie-Pierre Gagnon, Djesika D. Amendah

**Affiliations:** École Nationale de Santé Publique, Ouagadougou, Burkina Faso; Faculté Des Sciences Infirmières, Université Laval, Pavillon Ferdinand-Vandry, 1050, Avenue de la Médecine, Quebec, G1V 0A6 Canada; International Health Program, National Yang-Ming University, 155, Sec 2, Linong St, 112 Taipei, Taiwan; African Population and Health Research Center, APHRC Campus, 2nd Flore Manga Close, Off Kiwara Road, PO Box 10787-00100, Nairobi, Kenya

**Keywords:** Burkina Faso, Urban, Healthcare utilization, For-profit, Not-for-profit, Public, Expenditure, Out-of-pocket expenditure

## Abstract

**Background:**

Conventional wisdom suggests that out-of-pocket (OOP) expenditure reduces healthcare utilization. However, little is known about the expenditure borne in urban settings with the current development of the private health sector in sub-Saharan Africa. In an effort to update knowledge on medical expenditure, this study investigated the level and determinants of OOP among individuals reporting illness or injury in Ouagadougou, Burkina Faso and who either self-treated or received healthcare in either a private or public facility.

**Methods:**

A cross-sectional study was conducted with a representative sample of 1017 households (5638 individuals) between August and November 2011. Descriptive statistics and multivariate techniques including generalized estimating equations were used to analyze the data.

**Results:**

Among the surveyed sample, 29.6 % (n = 1666) persons reported a sickness or injury. Public providers were the single most important providers of care (36.3 %), whereas private and informal providers (i.e.: self-treatment, traditional healers) accounted for 29.8 and 34.0 %, respectively. Almost universally (96 %), households paid directly for care OOP. The average expenditure per episode of illness was 8404XOF (17.4USD) (median 3750XOF (7.8USD). The total expenditure was higher for those receiving care in private facilities compared to public ones [14,613.3XOF (30.3USD) vs. 8544.1XOF (17.7USD); *p* < 0.001], and the insured patients’ bill almost tripled uninsured (*p* < 0.001). Finally, medication was the most expensive component of expenditure in both public and private facilities with a mean of 8022.1XOF (16.7USD) and 12,270.5 (25.5USD), respectively.

**Conclusion:**

OOP was the principal payment mechanism of households. A significant difference in OOP was found between public and private provider users. Considering the importance of private healthcare in Burkina Faso, regulatory oversight is necessary. Furthermore, an extensive protection policy to shield households from catastrophic health expenditure is required.

## Background

Out-of-pocket (OOP) expenditure on healthcare imposes a significant burden on households facing a health crisis [1–3] and is a worldwide concern [4]. Significant OOP expenditure may lead households to a “financial catastrophe” in the absence of risk and payment pooling mechanisms or insurance.

In sub-Sahara Africa (SSA), generations of health system policy were implemented in the last three decades, prompted by a quest to balance improved health for populations and concern about financial equity in access to healthcare [5]. The *Bamako Initiative* approach was launched in 1987 to boost the primary care policy in African setting. This policy placed emphasis on cost recovery, community participation in facilities management, and sustainable essential drug-supply policy [6, 7]. Besides, other initiatives were tried such as community-based health insurance, a risk pooling scheme to render more affordable medical costs [8] or the pay-for-performance (or results-based financing) approach that targets both quantity and quality of healthcare delivery [9, 10]. All were implemented in the context of emerging private sector of care delivery. This sector is evidenced to contribute to healthcare delivery and to boost competition leading to quality improvement [11–13].

Similar to other countries in SSA, both private and public sectors coexist in Burkina Faso, shifting therefore from the long-standing free-of-charge policy in healthcare delivery to the user charge policy [14]. The rapid development of private sector in low and middle income countries (LMIC) resulted in diversified healthcare sources and options for consumers [15]. Meanwhile, healthcare costs incurred by patients rose [16], particularly for patients treated in the private sector [17]. However, private health sector is not efficiently regulated and monitoring policies are inoperative [18, 19]. The challenges to effective regulation derive from varied constraints: poor enforcement of health regulations, insufficient institutional capacity, lack of competition in the market, weak professional associations, or prevailing dual practice [20].

In Burkina Faso, the private health sector expanded rapidly and reinforced the traditional public health system, especially following the market liberalization (in 1991). With growth estimated at 104 % between 1997 and 2007, and at 28.5 % from 2007 to 2011 [21–23], a rise of the number of medical contacts was observed (0.50: 2008, 0.63: 2010, 0.78: 2013 [24]). As elsewhere, the private health sector in Burkina Faso is inclined towards curative care delivery [25]. This private sector consists of two distinct components with different philosophy: private-for-profit (PFP) and private-not-for-profit (PNFP). The first is defined as benefit-focused, while PNFP providers are philanthropic-oriented medical institutions (Faith-based and non-governmental organizations). Because of a longstanding partnership with government, PNFP providers receive support from the State in the form of trained personnel; and deliver similar services as in government facilities, including immunization and other public health programs associated with positive externalities [25].

As in other LMICs [26], in Burkina Faso, OOP is the principal healthcare payment scheme [26]. Although approximately 12.5 % of the state budget is allotted to health, fewer than 2 % of people are insured [27] and the health system still greatly depends on external funding (36.2 % in 2010 [28]). Despite the context of emergence and development of private sector in urban areas, scanty evidence exists on the level, distribution, and determinants of OOP expenditure in those areas. Yet, most studies have investigated rural settings [29–32], while urban areas have become home to numerous therapeutic systems from varied ownership and this expansion will continue [33]. This study sought to address those gaps in Ouagadougou by investigating: (a) the level of OOP expenditure on healthcare, (b) the distribution of OOP based on its primary components and on the ownership of healthcare facilities, and (c) the proximate determinants of OOP.

## Methods

### Study design and setting

A cross-sectional survey was conducted in the 30 administrative sectors (ASs) of Ouagadougou, the capital city of Burkina Faso from August to November in 2011. Ouagadougou is home to approximately 2 million inhabitants and has the lowest poverty level in the country (28.3 % versus national average of 46.7 %) [29]. It has an extensive number of both public and private health facilities (10 % of the public health facilities and 60.3 % of the private health facilities of Burkina Faso) [34, 35].

### Sample size and sampling procedure

The population for this study came from a doctoral dissertation project entitled “Health-Care-Seeking Patterns in the Emerging Private Sector in Burkina Faso.” Because of the absence of a households list, the simplified general method for cluster-sample surveys in developing countries [36] was implemented. To strengthen the statistical power and maximize the representativeness, the selection of the clusters (streets) used the cardinal point system (South, North, West, and East in random order) applied in each of the 30 ASs. A two-stage clustered sampling was implemented based on the city map to randomly select (without replacement) the streets in the individual AS (primary sampling unit). From each selected street, starting from the first entry point (South of the AS), a skipped interval was applied to map out the households to be surveyed (secondary sampling unit). The number of households per AS was defined according to a probability-proportional-to-size (PPS). The sampling procedure is exhaustively detailed elsewhere [37]. The number of households was obtained from the Burkina Faso National Institute of Statistics and Demographics (NISD). Based on studies of its kind [38–42], 1600 households were retained for the main project. For the purposes of this study, only households residing for 6 months (at least), in which at least one member had experienced any morbid event in the 30 days prior to the survey (n = 1025) were considered.

### Data collection procedures

Data were collected through an interviewer-based questionnaire. The survey adhered to the Demographic and Health Surveys (DHS) strategy which represents a standard in the field. Oral consent was sought from the household head on behalf of interviewees. In each household, the head and spouse (if any) were met separately for a face-to-face interview. In the case of a discrepancy, the household wife’s information was retained, assuming she is more aware of illnesses occurring in the household. Information on members who felt sick or were injured was recorded according to the “three-stage decision scheme” to portray health status and healthcare used [43]. First, it was asked whether any morbid event happened in the preceding 30 days. If any, then information was collected on the action taken, and finally, the type and name of provider sought, and the expenditure incurred. Six trained data collectors were assigned a fixed number of AS for this field survey. Supervision was insured by the principal investigator (PI) (BI). Each household head received a symbolic participation gift (battery-powered flashing pen) afterwards but did not have any prior knowledge of the gift to avoid undue influence.

### Data collection instrument and quality control

A structured questionnaire inspired by those developed and used in Burkina Faso’s DHS was employed. The questionnaire was content-validated, forward into French and backward translated, and finally pilot-tested with 32 households. The questionnaire included three sections. The first section covered sociodemographic variables: gender, age, occupation, education, filiation, marital status, and insurance coverage. The second covered information about the therapeutic action and type of provider consulted. The last module included the itemized financial cost of treatment.

As certain respondents used nicknames to designate facilities, further details were sought to match the designated provider with the Ministry of Health Department of the Private Sector master list. Onsite verifications were performed to obtain complementary information on facilities not officially listed (unlicensed or recently registered). For quality control, the PI re-interviewed 2 % of households with a short version of the questionnaire and scrutinized each questionnaire before data were entered into the Census and Survey Processing package (CSPro), version 4.0.

### Definition of variables

The dependent variable was expenditure recorded in West African CFA francs (XOF) (US$1 = 481.5XOF [44]). Explanatory variables consisted of gender, health insurance, the relationship with the household head, and age. Three other variables were specific to adults: education, marital status, and occupation. Regarding providers, the formal provider is the one providing western-based care. This included public facilities [primary healthcare centre (PHC), district hospital (DH), and teaching hospital (TH)] and formal private facilities (PFP and PNFP). Informal sources included self-treatment, traditional healers, and marabouts.

### Data analysis

Only expenditure related to acute health experience was analysed to better portray the unplanned characteristic of the financial burden. Therefore, chronic diseases, planned surgeries, and hospitalized cases were excluded. The market price of medicine was considered for any home treatment. Descriptive analysis was conducted and analysis of variance (ANOVA) or *t* test (as appropriate) fitted to assess how significant the difference in mean expenditure was. The median expenditure is also presented for its robustness. Because of positively skewed expenditure data, the log of expenditure was computed to better approximate a normal distribution for inference. A p value of less than 0.05 was considered significant.

Generalized estimating equations (GEE) was implemented to account for the household level clustered pattern of the data. More than one person in a household could incur expenditure and health-seeking decision within household is probably correlated. This approach is recommended for correlated data [45]. On the other hand, behaviour and expenditures in different households are probably independent. All the statistical analyses were performed using STATA, version 12.1 (Stata Corp., College Station, TX, USA) and SPSS, version 21 (SPSS inc., IL: Chicago, USA).

### Ethical considerations

Ethical clearance was granted from the Burkina Faso National Research Ethics Committee upon an examination and an oral presentation of the proposal (#2011-11-82). Additional permission was obtained from the Ouagadougou city council. Prior to each interview, respondents were clearly informed of the voluntary nature of their participation and could decline their consent at any time. Data collected was anonymized at all levels of the study.

## Results

### Characteristics of the study population

**Fig. 1:**
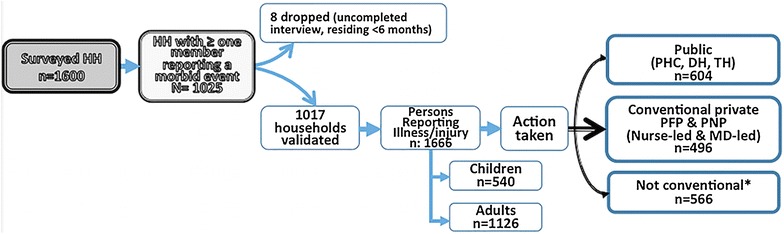
Healthcare providers sought. *Asterisk* include self-treatment traditional healer and other not formal provider. *PHC* primary healthcare center, *DH* district hospital, *TH* teaching hospital, *HH* household, *PFP* private for-profit, *PNFP* private not-for-profit, *MD* medical doctor

The survey covered 1025 households of which 8 were discarded for incomplete information. The final sample included 5633 persons, of whom 1666 (29.6 %) reported a morbid event. These 1666 persons constituted the analytical sample of interest (Fig. 1). Among them, 940 (56.5 %) were women, and 1126 (67.6 %) were aged 15 years or older. More than half of sample (n = 918, 55.1 %) were sons-daughters or grandsons-daughters. Among the sick adults (≥15 years), 808 (72.1 %) completed at least primary education and only 158 (14.1 %) held a formal job. Sixty-five (3.9 %) of the participants had a health insurance plan. Table 1 indicates that among the persons who reported a sickness, 1100 persons (66.0 %) had sought care from a formal health provider (public or private). In terms of morbidity patterns, malaria diagnosed at a health facility and presumptive malaria was the cause for illness for 1087 (65.3 %) persons. The other 579 (34.7 %) respondents who were sick reported a wide range of pathologies, including injuries and other infectious medical conditions (data not shown). Finally, the logistic regression test of the likelihood of reporting sickness showed that households with fewer sick members report more morbid events: 1–2 persons (OR: 2.43, 95 % CI: 1.88; 3.14), 2–3 persons (OR: 1.29, 95 % CI: 1.12; 1.48) (data not shown).

**Table 1:** Characteristics of participants reporting disease/injury (n = 1666)

Characteristics	n	Mean
Reporting disease/household		
1 person	626	37.6
2–3 persons	733	44.0
>3 persons	307	18.4
Age group		
Children (<14 years)	540	32.4
Adults (> 5 years)	1126	67.6
Gender		
Female	940	56.5
Male	725	43.5
Missing	1	–
Relationship with household head		
Household head and spouse	602	36.1
Son-daughter and grandson-daughter	918	55.1
Other household member	146	8.8
Marital status among adults		
In union	520	42.1
Single	616	49.9
Divorced/separated/widow	99	8.0
Missing	2	–
Education level among adults		
No formal education	312	27.9
Primary level	154	13.7
Secondary level	518	46.3
University	136	12.1
Missing	6	**–**
Occupation among adults		
Formal (public, para-public and private)	158	14.1
Informal private	249	22.3
Not in labour (retired, students…)	711	63.6
Missing	8	**–**
Insurance		
Not Insured	1601	96.1
Insured	65	3.9

### Health care expenditure by main characteristics and type of providers sought

To adhere to unplanned characteristic of the financial burden, 24 participants were excluded because they either consulted for a chronic disease, or were hospitalized, or had a planned surgery. Table 2 details health expenditure by main characteristics and type of providers. The mean total expenditure was 8404XOF (17.4USD) but age and insurance coverage significantly affect expenditures. Sick adults (≥15 year) spent 40 % more than children [9301.4XOF (19.3USD) vs. 6602.3XOF (13.7USD); *p* < 0.0001]. Insured persons spent almost three times as high as that of uninsured persons [22,537.4XOF (46.8USD) vs. 7824.1XOF (16.2USD); *p* < 0.001], but gender did not significantly affect spending (*p* = 0.156).

**Table 2:** Mean total medical expenditure by selected variables (n = 1573)

Characteristics	N	Mean (SD)	*p* *value* ^a^	Median
Age, year				
0–4	185	7008.41 (10,966.79)	<0.001	3500.00
5–14	338	6380.08 (11,112.00)		2500.00
15–64	984	9211.79 (15,833.49)		4000.00
>64	66	10,637.86 (14,631.00)		5075.00
Gender				
Female	888	8708.06 (14,648.42)	0.156	4100.00
Male	684	8020.14 (14,123.63)		3175.00
Filiation				
Household head and spouse	55	11,083.36 (18,691.55)	<0.001	4500.00
Son-daughter and grandson-daughter	883	6646.12 (10,679.26)		3013.00
Other members	137	8919.02 (13,993.48)		4500.00
Insurance status				
Not insured	1511	7824.09 (13,444.66)	<0.001	3500.00
Insured	62	22,537.42 (25,916.23)		13,600.00
Type of provider				
Self-treatment	529	2723.82 (3818.42)	<0.001	1200.00
Public	573	8544.06 (14,217.96)		4200.00
Private	471	14,613.34 (18,919.88)		8750.00
Type of disease experienced				
Malaria	1075	5671.66 (9171.64)	<0.001	2900.00
Otherwise	498	14,302.19 (20,610.18)		6750.00
Total	1573	8404.02 (14,418.53)		3750.00

^a^p value of log-expenditure

Table 3 details the mean total expenditure by type of provider. Of the 1573 persons who reported their expenditures, those treated at TH incurred the highest mean expenditure [mean: 29,270.8XOF (60.8USD), median: 19,900XOF (41.3USD)], followed by those treated in doctor-led PFP facilities [mean: 26,937.2XOF (55.9USD), median: 18,800XOF (39.0USD)]. Persons treated at doctor-led PNFP incurred expenditure that are about half those of persons treated in doctor-led PFP [mean: 13,044.5XOF (27.1USD), median: 6200XOF (12.9USD)]. Furthermore, persons who received care at PNFP led by a nurse had the lowest expenditure among those who received care in the formal system [mean: 5952.1XOF (12.3USD), median: 4100XOF (8.5USD)]. Participants who commenced the treatment at home (649 out of 785) reported an additional mean cost of 1910.4XOF (3.9USD) [median: 700XOF (1.5USD)], and most of them, (587 persons, 90.4 %) reported a malaria case. Overall, those who received care from other sources (i.e., priest, home visits) had 14 % lower expenditure than the sample mean [mean: 7188.8XOF (14.9USD), median: 6000XOF (12.5USD)] and self-medication appeared to be the least expensive option [mean: 2458.7XOF (5.1USD), median: 1000XOF (2.1USD)].

**Table 3:** Mean total medical expenditure by type of providers visited (n = 1573)

Providers	n	Mean (SD)	*p* *value* ^a^	Median
Formal providers				
Private	471	14,613.34 (18,919.88)	<0.001	8750.00
Public	573	8544.06 (14,217.96)		4200.00
Formal providers (break-down)				
Private for-profit doctor	111	26,937.21 (26,030.78)	<0.001	18,800.00
Private for-profit nurse	127	10,015.28 (10,553)		7500.00
Private not-for-profit doctor	174	13,044.49 (17,609.24)		6200.00
Private not-for-profit nurse	59	5952.10 (5303.422)		4100.00
Primary healthcare centre	412	6104.27 (9750.91)		3500.00
District hospital	137	12,250.26 (17,266.58)		8150.00
Teaching hospital	24	29,270.83 (30,475.27)		19,900.00
Informal providers				
Self-treatment	501	2458.68 (3261.88)	<0.001	1000.00
Traditional healer	1	15,000 (-)		15,000.00
Other (priest, praying group, other)	27	7188.85 (7976.57)		6000.00

^a^p value of log-expenditure

### Itemized health expenditure by facility ownership

Table 4 presents expenditure by item stratified by type of provider. Among the 398 participants who recalled the amount of expenditure by item, the single most expensive expenditure item was drug [mean: 10,256.8 (21.3USD)], median [7500 (15.5 USD)]. Laboratory/imagery appeared to come close second for the amount spent [mean: 8965.1XOF (18.6USD), median: 5333.5XOF (11.1USD)].

**Table 4:** Mean item cost incurred in visiting private (PFP, PNFP) and public sources of care

Variable	n	Combined providers	n	Private	n	Public	p value^a^
		Mean (median|min–max)		Mean (median|min–max)		Mean (median|min–max)	
Visit fee	361	1629.64 (500|100–20,000)	191	2328.79 (1000|100–20,000)	170	844.12 (300|100–15,000)	<0.001
Drug	365	10,256.88 (7500|150.0–10,000)	192	12,270.54 (10,000|150–100,000)	173	8022.06 (6000|400–50,000)	<0.001
Lab/test	85	8976.47 (5333|100–100,000)	54	8714.20 (6000|500–50,000)	31	9433.32 (5000|100–100,000)	0.208
Transport	188	1974.47 (1200|50–30,000)	107	2258.73 (1450|50–30,000)	81	(1598.97 (1000|50–24,500)	0.008
Other fees	22	1677.23 (666|100–10,000)	10	1029.90 (666.5|100–5100)	12	2216.67 (450|100–10,000)	0.488

^a^p value of log-expenditure

*PFP* private-for-profit, *PNFP* private-not-for-profit

A comparison by providers suggests that patients in private facilities paid about 50 % more for their drugs than those in public facilities [mean: 12,270.5 XOF (25.5USD) vs. 8022.1 XOF (16.7 USD), *p* < 0.001]. Similarly, patients in private facilities paid about 100 % more for consultation fees than those in public facilities [mean: 2328.8 (4.8 USD), median: 1000 (2.1) vs. 844.1 (1.7 USD), 300 (0.6 USD), *p* < 0.001]. Consultation fees covered a wide range, from 100XOF (0.2USD) (in PHC), to 20,000XOF (41.5USD) (specialists). In both private and public providers, in average, adults spent about 69 % more than children [mean: 16,609.7XOF (34.5USD) vs. 9837.9XOF (20.4USD), *p* < 0.001; not shown].

Finally, those who visited public or private facilities, spent less for a malaria case treatment than for any other category of illness (means: 5671.7XOF [11.8USD] vs. 14,302.2XOF [29.7USD], *p* < 0.001).

### Proximate determinants of OOP expenditure

The multivariate results (Table 5) indicate that age, gender, and relationship with the household head were not significant predictors of health expenditure. Households with fewer sick members (≤3) tended to spend more (model 1). Insured persons spent 56 % (model 1) and 51 % (model 2) more than uninsured. Furthermore, patients seeking care in private facilities spent 48 % more than those in public facilities (Model 1) (95 % CI = 0.27; 0.69, *p* < 0.001). When using participants who treated themselves as reference, those who attended public facilities spent 97 % more (95 % CI = 0.67; 1.27, p < 0.001) and those who attended private 141 % more (95 % CI = 1.11; 1.71, *p* < 0.001) (Model 2). Finally, malaria patients experienced less of a financial burden compared to the rest of medical conditions (*p* < 0.001).

**Table 5:** Log-linear generalized estimating equations of expenditure on health

	Model 1				Model 2			
	Coef.	SE	95 % CI	*p value*	Coef.	SE	95 % CI	*p* *value*
Age, year (>64 = ref)								
0–4	−0.20	0.21	−0.60; 0.21	0.348	−0.17	0.16	−0.49; 0.15	0.289
5–14	−0.08	0.19	−0.45; 0.29	0.672	−0.13	0.14	−0.41; 0.15	0.371
15–64	−0.03	0.16	−0.034; 0.29	0.870	−0.06	0.13	−0.30; 0.19	0.653
Gender (male = ref)								
Female	0.00	0.06	−0.13; 0.12	0.949	−0.01	0.05	−0.11; 0.09	0.882
Household size (>3 persons = ref)								
1 person	1.18	0.28	0.63; 1.74	<0.001	0.86	0.24	0.38; 1.34	<0.001
2–3 persons	0.86	0.32	0.22; 1.49	0.008	0.63	0.27	0.10; 1.16	0.020
Filiation (other member = ref)								
Household head and spouse	−0.09	0.11	−0.32; 0.13	0.415	0.14	0.09	−0.04; 0.32	0.140
Son-daughter/grandson-daughter	−0.13	0.13	−0.40; 0.13	0.333	0.02	0.10	−0.017; 0.21	0.820
Type of disease (other conditions = ref)								
Reporting malaria	−0.86	0.12	−1.09; −0.63	<0.001	−0.64	0.11	−0.85; −0.43	<0.001
Insurance (uninsured = ref)								
Insured	0.56	0.22	0.13; 1.00	0.010	0.51	0.17	0.17; 0.85	0.003
Type of provider^a^								
Self-treatment								
Public					0.97	0.15	0.67; 1.27	<0.001
Private	0.48	0.11	0.27; 0.69	<0.001	1.41	0.15	1.11; 1.71	<0.001
Total observations (n)				1044				1573

All estimates are adjusted for each covariate for the effects of the other covariates

^a^In model 1, we exclude persons who self-treated and used public provider as reference

^a^In model 2 we included the entire sample and used self-treatment as a reference for public and private provider choice

*CI* confidence interval, *SE* standard error

## Discussion

Three crucial results were found. First, residents of Ouagadougou received care from a variety of providers. Second, while the mean OOP expenditure was 8404XOF [17.4USD], persons who visited private providers paid, on average 50 % more than those treated in the public sector. Likewise, insured patients appeared to incur even higher expenditure. Finally, medication was the single most expensive component of the OOP expenditure.

The mean OOP in this study represent 27 % of the minimum legal monthly wage of 30,684XOF [63.7USD]. In addition, a single morbid episode treated by PFP doctor costs 115.40 % of the monthly per capita expenditure of Ouagadougou residents [46]. In other words, PFPs are beyond the reach of modest families in Ouagadougou. Similar results have been found elsewhere. In a single study in Vietnam, Thuan et al. [47] indicated that annual health expenditure amounted to 247.3 Vietnamese Dong (VND) in formal private facilities compared to 59.9 VND in a public reference hospital. In one recent study on malaria care seeking for children under 5 years old in Uganda, Orem and al [48]. observed that, medicine cost (2.3USD) less than consultation (3.3USD) and hospitalization (7.6USD). The private providers were the preferred option, although, the odds of incurring OOP were 13.4 times higher than the odds for a child who went to a public facility. The literature suggests that possible reasons of higher expenditures in private facilities include the fact that certain patients trust private providers more [49], appreciate their interpersonal quality [50], and perceived improved quality of services [51]. Certain authors have suggested that patients tend to patronize private providers for moderate or acute health conditions [50, 51]. In an early research in Ouagadougou, Beogo et al. [37] suggested that the utilization of the PFP health facilities was predicted by enabling factors that include insurance coverage, high education level, and holding a good job. An extensive insurance plan might help reduce families’ exposure to OOP, which might ultimately lead to catastrophic health expenditure and impoverishment.

Surprisingly and interestingly, insured patients paid high financial toll. Two hypotheses were set: (1) the behaviour of practitioners and (2) the type of provider that insured patients favoured might explain this fact. First, in Ouagadougou, insured persons are among the wealthiest and most hold private insurance plans which are 80–100 % refund-based. Indeed, medical bill inflation correlation with insurance is a well-known phenomena [52]. However, little is known on difference in prescription in literature. It could be argued that practitioners behave discriminatorily with insured patients. Since it is assumed that they will be refunded, costly diagnostic tests, treatments, and/or brand medicines are prescribed, even though generic medicines are available. The third party will help pay the higher bill. Such a supplier-side induction behavior could be majored by the demand-side one, aggrandizing therefore the bill. The second argument is based merely on the high burden incurred by PFP. Apart from the user charge that are the highest in the market—also evidenced in this study—insured patients are prompt to patronize PFP [53, 54], known to bill comparatively higher [17]. The present study finding on health insurance is relevant and important as the central government develops a national insurance. It provides insight and encourages policy makers to be mindful.

In Burkina Faso, the government owns an extensive network of PHCs—the first line of health facilities—which are the pillar of primary care delivery. They are easily accessible geographically, state-subsidized, and offer an integrated service that includes preventive care (vaccination, infant check-up, etc.), basic curative care, and generic drug dispensation at a nominal price. In addition, primary healthcare facilities are mainly staffed by nurses or midwives whose salaries are lower than those of doctors. The cost to the State to operate public facilities is therefore lower than that of the private sector, translating into lower expenditure for patients.

The study ranked healthcare providers according to the expenditure incurred by patients receiving care therein. PFP facilities are the most expensive, followed by PNFP, and public providers, excluding tertiary hospitals. The PNFP and public providers are probably more affordable for patients because of their organizational structure: patients are treated by nurses (1st line) who manage common conditions and refer only complex cases to doctors (2nd line) or specialist (3rd line). On the contrary, in the private sector, in doctor-led PFP especially, doctor sees all patients and the cost structure leans toward price maximization.

The propensity to self-treat raises the issue of financial access, perceived severity of the disease, or resilience to malaria. Geographical access is unlikely be an obstacle in Ouagadougou, where the mean radius to any facility is the lowest in the country (1.7 vs. 6.4 km). Such a high proportion might be strongly associated with seasonality [55].

In the current study, medication was the single most expensive element of the household healthcare bill. Similar results have been found in Burkina and elsewhere. The NISD [46] reported that medications accounted for 75.6 % of the health expenditure by households in 2003. In Chad, medication took up 64.5 % of the total patient bill for medical visits in the capital city [56]. That item accounted for 58–70 % of the total treatment costs per person in urban Nigeria [57]. Nguyen [17] clearly outlined the weight of medications in private medical bills in one Vietnamese study: patients in the private sector were likely to be prescribed 4.1 drug items on average, or 3.8 drug items in a tertiary institution, with more injections. In Tajikistan, Tediosi et al. [58] observed that 76.7 % of visits lead to a medicine prescription. In one ecological study in low income countries, medicine was the largest component of OOP expenditure for outpatient services in both public and private facilities (57 and 45 %; [59]).

Two points have emerged from the sociodemographic determinants of expenditure from the present study. First is the parallel upward trend between age and the amount paid for care received. This pattern concurs with the results of Steinhardt et al. [60], except that in the present study, the correlation is irrespective of the provider type. Besides, it appeared that the fewer the number of persons in a household, the greater the likelihood of reporting a disease. This result might be explained either by the recall bias or selection bias, because substantial differences exist in reported illness or injury and the actual illness experienced [61], due to family wealth [62], even for child healthcare [63]. The recall period of 30 days was used as suggested by Heijink et al. [64]. But 30 days might be too long since a sizeable proportion of interviewees is illiterate (41.6 %), and might not be able to record their expenditure. This might explain why a number of persons could not recall item costs in the total bill. Secondly, expenditure was not relates to quality, severity of illness, and other services variables. Finally, the study targeted direct costs and did not address indirect cost such as loss of income. In particular, information on queuing and waiting time was not collected. That could be longer in public facilities, meaning that while direct costs were lower for care received there, the indirect cost (time lost not only for the sick person but also for the caregiver if any) might be higher.

## Conclusion

The present study provides a snapshot of medical expenditure and its determinants in a SSA urban setting with a diverse provision of care. The present study emphasizes the high level of OOP expenditure for patients since Burkina Faso applies a cost recovery policy and the country does not have an extensive insurance plan. A significant difference on expenditure was observed by ownership of the facilities with care at private providers causing the highest expenditures. Furthermore, medication represented the largest share of the medical expenditure across providers. Finally, this paper highlights the importance of the private sector as a key player in the health system. Further, findings from this paper could inform policy in the ongoing national healthcare insurance debate in Burkina Faso and elsewhere. The national insurance plan in Burkina Faso is currently at the design stage, and if implemented, may help improve healthcare access by covering expenditure incurred in both public and private facilities and by protecting financially vulnerable households.

## Abbreviations

AS: administrative sector
DH: district hospital
GEE: generalized estimating equations
LMIC: low and middle income countries
OOP: out-of-pocket
PHC: primary healthcare center
PFP: private for profit
PNFP: private not-for-profit
SSA: sub-Sahara Africa
USD: US dollar
TH: teaching hospital
XOF: Franc CFA (West Africa)
ISO: currency code
